# Archean continental crust formed by magma hybridization and voluminous partial melting

**DOI:** 10.1038/s41598-021-84300-y

**Published:** 2021-03-04

**Authors:** Juan David Hernández-Montenegro, Richard M. Palin, Carlos A. Zuluaga, David Hernández-Uribe

**Affiliations:** 1 Department of Geosciences, Universidad Nacional de Colombia, Bogotá, Colombia; 2 Department of Earth Sciences, University of Oxford, South Parks Road, Oxford, OX1 3AN UK; 3 Department of Earth and Environmental Sciences, University of Michigan, 1100 North University Avenue, Ann Arbor, MI 48109-1005 USA; 4 Present Address: Division of Geological and Planetary Sciences, California Institute of Technology, 1200 East California Boulevard, Pasadena, CA 91125 USA

**Keywords:** Geochemistry, Geodynamics, Geology, Petrology

## Abstract

Archean (4.0–2.5 Ga) tonalite–trondhjemite–granodiorite (TTG) terranes represent fragments of Earth’s first continents that formed via high-grade metamorphism and partial melting of hydrated basaltic crust. While a range of geodynamic regimes can explain the production of TTG magmas, the processes by which they separated from their source and acquired distinctive geochemical signatures remain uncertain. This limits our understanding of how the continental crust internally differentiates, which in turn controls its potential for long-term stabilization as cratonic nuclei. Here, we show via petrological modeling that hydrous Archean mafic crust metamorphosed in a non-plate tectonic regime produces individual pulses of magma with major-, minor-, and trace-element signatures resembling—but not always matching—natural Archean TTGs. Critically, magma hybridization due to co-mingling and accumulation of multiple melt fractions during ascent through the overlying crust eliminates geochemical discrepancies identified when assuming that TTGs formed via crystallization of discrete melt pulses. We posit that much Archean continental crust is made of hybrid magmas that represent up to ~ 40 vol% of partial melts produced along thermal gradients of 50–100 °C/kbar, characteristic of overthickened mafic Archean crust at the head of a mantle plume, crustal overturns, or lithospheric peels.

Archean TTGs are composite suites of variably deformed granitoids that comprise two-thirds of Earth’s earliest continents^1^. Based on experimental and petrological evidence, it is generally accepted that most TTGs crystallized from magmas generated via partial melting of a hydrated mafic protolith^2,3^; however, fractionation of mantle-derived melts into sodic granitoids have also been considered a plausible alternative^4–6^. TTGs are characterized by magnesium numbers (Mg#) ~ 0.2–0.6 (averaging ~ 0.43), a potassium/sodium ratio (K_2_O/Na_2_O) less than 0.8, and a metaluminous character (aluminum saturation index, ASI: ~ 1.0)^1,4,5,7^. The source rock mineralogy during anatexis imparts a diagnostic trace-element signature on TTGs, which allows inferring the *P–T* conditions at which their parental melts equilibrate^1,3^. Accordingly, TTGs have been historically classified into a high-pressure group showing high Sr/Y and La/Yb (garnet-rich, plagioclase-free residuum), a low-pressure group with low Sr/Y and La/Yb (plagioclase-rich, garnet-free residuum), and a transitional medium-pressure group, where both plagioclase and garnet were present in the residuum in variable amounts^1,3^. How TTGs acquired these particular signatures is still a matter of debate. In general, petrological models invoke partial melting of subducted oceanic slabs in convergent plate margins akin to adakite formation on the modern-day Earth (e.g.,^8–11^) or partial melting of basaltic crust in non-plate tectonics regimes (e.g.,^12–15^). These models, however, do not always reproduce some compositional characteristics of TTGs observed in nature (e.g., low K_2_O/Na_2_O and high Mg#), which hinders a full understanding of how Earth’s first continents formed and have subsequently evolved through time. In this work, we integrate petrological and trace element modeling to calculate melt fractions generated during metamorphism of Archean basaltic crust under open and closed system conditions. We show that following voluminous partial melting, accumulation and hybridization of multiple melt fractions, which would most likely occur during vertical ascent through the crust, was necessary to generate felsic plutons matching the average composition of Archean TTG suites exposed on Earth today.

## Results

### Calculated phase equilibria

The enriched Archean tholeiite (EAT^16^) used as the protolith in this work has major- and trace-element signatures akin to modern-day oceanic island basalts (Supplementary Table 1), and is widely considered as a suitable source rock for the generation of Archean TTGs^2^. Metamorphism and partial melting were examined along a range of geotherms representing burial and heating paths experienced during prograde metamorphism. These linearized thermal gradients (50, 75, and 100 °C/kbar) characterize the wide range of metamorphic *P–T* conditions reported from Archean terranes before the emergence of plate tectonics^17,18^, and correlate with metamorphic conditions expected at the base of thick oceanic crust^18,19,20^. The H_2_O content was fixed to allow minimum water-saturation at the point of initial melting along each of these geotherms, which provides a lower bound on the volume of melt generated during metamorphism at water-saturated conditions (Supplementary Table 2). With open-system conditions, melt loss events (MLE) were considered to occur each time the amount of melt reached a critical threshold of 20 vol%. This volume threshold was chosen based on rheological studies on partially molten amphibolites and felsic magmas (e.g.,^21,22^), which indicate that melt segregation and transport can occur when ~ 20 vol% melt or more is present in the system.

For comparison, open system calculations were also conducted considering water-undersaturated conditions (1.0 wt% H_2_O) and water-fluxed melting (3.0 wt% H_2_O) with partial melts produced along the 75 °C/kbar thermal gradient (Supplementary Table 2). We also considered the effect of varying the critical melt fraction at which melt is extracted from the source since melt loss and melt segregation are unlikely to occur at a fixed melt volume due to the inherent heterogeneities of the system^22,23^. Accordingly, we modeled two additional scenarios with melt extraction occurring at critical thresholds of 15 and 25 vol% melt. In both cases, calculations were carried out assuming minimally water-saturated conditions at the intersection between the solidus and the 75 °C/kbar gradient.

**Figure 1:**
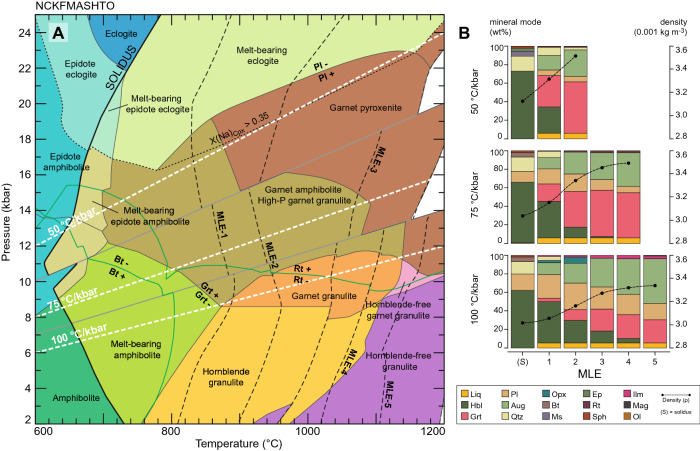
(**A**) Calculated *P–T* phase diagram for EAT showing the effects of melt fractionation during prograde metamorphism. Each melt loss event initiates at ~ 20 vol% of melt (black dashed lines) and phase relations calculated up-grade of this point are for a melt-depleted residuum. Geotherms are shown as dashed white lines. Dotted line marked X(Na)_Cpx_ > 0.35 divides high-pressure amphibolite and granulite-facies assemblages from eclogite-facies assemblages based on clinopyroxene composition. (**B**) Modal mineralogy present in the residuum at the point of first melting and each MLE afterward. Dotted black lines represent the density of the residuum. Calculations were performed in the Na_2_O–CaO–K_2_O–FeO–MgO–Al_2_O_3_–SiO_2_–H_2_O–TiO_2_–O_2_ (NCKFMASHTO) compositional system using Theriak-Domino software^24^ (Version 11.02.2015). Mineral abbreviations are based on Whitney and Evans^25^.

Figure 1 shows the summary *P–T* assemblage diagram and modal mineralogy for EAT at subsolidus conditions and after each event of melt loss (for the un-simplified version of this pseudosection see Supplementary Figure 1). The amount of water required to minimally saturate the protolith at its solidus decreases toward high-pressure conditions, resulting in suppressed partial melting at eclogite- and epidote–amphibolite-facies conditions and higher solidus temperatures^13^. By contrast, the H_2_O content that allows minimal fluid saturation prior to the onset of partial melting is higher at intermediate to low pressures (~ 8–14 kbar) where the modal proportion of subsolidus amphibole increases (Fig. 1B). This enhances melt productivity chiefly by amphibole dehydration melting and reduces the solidus temperatures for the 50 and 75 °C/kbar geotherms (at ~ 640 °C). Melt productivity increases towards higher geothermal gradients such that more melt extraction events—and larger accumulated melt volumes—result at amphibolite to granulite facies (Fig. 1).

The stability of plagioclase marks the limit between eclogite and high-pressure amphibolite/garnet pyroxenite facies residue at pressures ~ 18–20 kbar above 800 °C. Garnet is stable at pressures as low as ~ 8 kbar at high temperatures and is absent from all subsolidus assemblages along the modeled thermal gradients. The mode of this phase increases with progressive melting and melt extraction. As the thermal gradient increases, the stability field of hornblende extends toward higher temperatures so that it is consumed at ~ 900 °C and ~ 1100 °C along the 50 and 100 °C/kbar geotherms, respectively. Moreover, the presence of orthopyroxene constrains granulite-facies conditions to pressures below ~ 10–12 kbar and temperatures above ~ 800 °C, being in equilibrium with garnet at low pressures along the 100 °C/kbar geotherm (Fig. 1).

The proportion of solid phases in the subsolidus region remains the same as the water content increases above minimally saturated conditions (Supplementary Figure 2). In this case, initial partial melting quickly consumes water and plagioclase, leading to a first MLE at relatively low temperature, after which partial melting continues via dehydration of biotite and hornblende (Supplementary Figure 2). Minimally water-saturated and water-excess conditions at the solidus produce four and five MLE, respectively, along the 75 °C/kbar geotherm. By contrast, fewer MLE occur as the content of water decreases from water-fluxed to water-absent melting conditions, which also causes the solidus position to shift toward higher temperatures (Supplementary Figure 2). Water-undersaturated conditions result in lower stability of hydrous phases such as biotite and hornblende, while the stability field of garnet is extended to lower temperature. The proportion of plagioclase also increases with lower water content but remains relatively constant after partial melting and melt extraction. Partial melting occurs via amphibole dehydration melting, which also produces garnet and clinopyroxene (Supplementary Figure 2).

The proportion of solid phases is identical for all the considered melt extraction thresholds along the 75 °C/kbar with minimally water-saturated solidus conditions (Supplementary Figure 3). Variations in the critical melt fraction mainly affect the temperature at which melt extraction occurs and the total number of MLE. Consequently, five MLE take place with a melt threshold of 15 vol%, four with 25 vol%, and only three with 25 vol% (Supplementary Figure 3).

### Melt compositions

**Figure 2:**
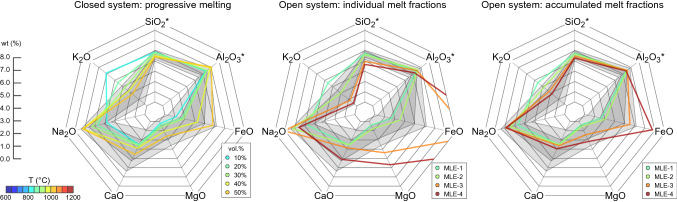
Major-element compositions for melts produced along a thermal gradient of 75 °C/kbar. Colors represent the temperature range at which each melt fraction equilibrated and separated from the source. Dark and light gray regions represent the mean composition of natural Archean TTGs within ± 1σ and ± 2σ^1^, respectively.

**Figure 3:**
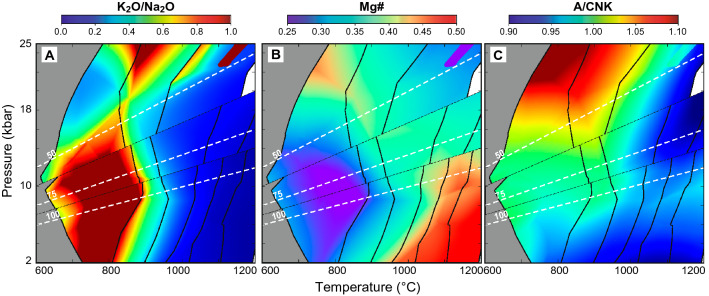
Color-maps showing the pressure–temperature (*P–T*) dependence on partial melt compositional characteristics. (**A**) K_2_O/Na_2_O, (**B**) Mg#: (Mg/Fe + Mg), and **C:** A/CNK: (Al/Ca + Na + K; ASI). Phase equilibria and MLE are the same as those shown in Fig. 1. Calculations were performed using Theriak-Domino software^24^ (Version 11.02.2015).

Representative major-element compositions of melts produced along the 75 °C/kbar geotherm are shown in Fig. 2. Figure 3 shows changes in key melt parameters for Archean TTGs across the *P–T* space. All calculated major-element melt compositions are given in Supplementary Table 3. In closed-system conditions, the melt fractions produced remain in equilibrium with the solid residuum and the initial equilibration volume is always the same. By contrast, melts generated under open-system conditions are considered to separate from their source during each MLE so that the composition of both the melt and the source is progressively changing with continued melting. We integrated the composition of each individual melt fraction to simulate magma mixing and accumulation as melts migrate toward upper levels of the crust. This was carried out by adding the elemental composition of consecutive melt fractions produced along a particular thermal gradient. Similarly, the composition of the system after melt loss was recalculated by subtracting the elemental composition of the melt, allowing a quarter of the produced melt to remain in the source. The melt compositions obtained in this work represent direct products of partial melting and are therefore only approximated compositions of felsic granitoids. In nature, initial partial melts would be more likely modified during magma emplacement in upper crustal levels due to fractional crystallization or mixing and assimilation of magmas from external sources.

The first melts produced have high SiO_2_ and K_2_O contents due to breakdown of biotite and/or muscovite during initial partial melting, although these components are less abundant in subsequent melt fractions (Fig. 2). As a result, partial melts produced prior to the first melt loss event have high K_2_O/Na_2_O ratios (Fig. 3A), which differ from most Archean TTG values and resemble more the composition of potassic granitoids^1,26^. Melt fractions with K_2_O/Na_2_O ratios in the range of natural Archean TTGs require the production of high melt volumes in a closed system (> 20 vol%) or the occurrence of multiple MLE (Figs. 2 and 3A). Likewise, the Fe^2+^/Mg proportion in the melts increases when garnet and pyroxene are absent from the residuum (or just present in small proportions), thus resulting in low Mg# for melts equilibrated at pressures below ~ 15–18 kbar and before the first melt extraction event (Figs. 1 and 3B). The production of melts with high Mg# requires high to intermediate pressures of melting, generation of substantial melt volumes (~ 20 vol% or more), or multiple MLE (Fig. 3B). Furthermore, substantial melting at high pressures is mainly controlled by breakdown of hydrated, Al-rich minerals such as hornblende, muscovite, and epidote (Fig. 1). As a result, partial melting above ~ 18 kbar produces strongly peraluminous melts with ASI values above the average of sodic TTG magmas (Fig. 3C).

Both water-fluxed and water-undersaturated melting produce initial melt fractions with high Mg# and K_2_O/Na_2_O ratio. However, the K_2_O/Na_2_O ratio is slightly lower with water excess conditions as partial melting consumes nearly all plagioclase prior to melt extraction (Supplementary Figure 2). The first MLE under water-saturated conditions occurs at ~ 733 °C with a thermal gradient of 75 °C/kbar and generates a melt composition with low FeO, MgO, and CaO contents, which then increase in subsequent melts. Water-fluxed melting produces between ~ 20 and ~ 50 vol% of hybrid melt fractions matching the major element composition of average TTGs (Supplementary Figure 4), which further supports the idea that fluids availability at relatively shallow depths may also produce TTG-like magmas with the ‘high pressure’ signature^27^. At water-undersaturated conditions, mixing of melt fractions generated during the first and second melt loss events is required to produce ~ 30 vol% of magmas resembling average TTGs (Supplementary Figure 4). In general, both water-undersaturated and water-fluxed melting can generate TTG-like compositions at temperatures above ~ 1050 °C, but water-fluxed melting produces ~ 20% more melt (Supplementary Table 3).

The effect of varying the critical melt fraction on the composition of hybrid melts generated at similar *P–T* conditions is negligible. However, initial partial melts generated with a critical threshold of 15 vol% are more enriched in K_2_O, which is expected as the concentration of incompatible elements in the melt increases at lower melt fractions. In the three cases, extracted melts have progressively lower K_2_O/Na_2_O ratio and higher FeO and MgO contents as partial melting proceeds (Supplementary Figure 5). Melt compositions in the range of natural TTG variations are generated at temperatures ~ 900 and ~ 1070 °C, representing ~ 20–40 vol% hybrid melts (Supplementary Table 3).

### Trace-element modeling

The trace-element composition of melts derived from metabasaltic rocks is strongly controlled by the abundance of garnet, plagioclase, rutile, amphibole, and pyroxene in the residuum^28,29^, which in this case represent more than 85% of the mineral assemblage (Fig. 1B). Other processes that may affect TTG geochemical signatures include assimilation-fractional crystallization^30^ (AFC) or early crystallization of peritectic phases^31^, although we do not consider these effects here. To study the trace-element evolution of the melt fractions obtained from phase equilibria, we carried out batch melting calculations based on mineral-melt partition coefficients as described in the Methods section ^12,28,32^.

**Figure 4:**
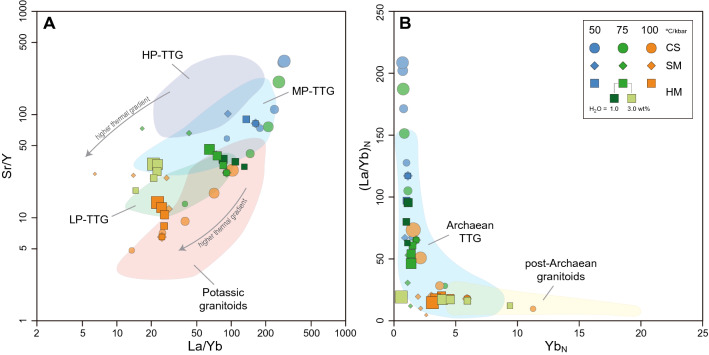
Bivariate plots for (**A**) Sr/Y vs La/Yb and (**B**) (La/Yb)_N_ vs Yb_N_ showing the geochemistry of calculated melts. Colored fields correspond to the regions defined by the high-, medium-, and low-pressure classification system for sodic TTGs and potassic granitoids from Moyen (2011). CS = Closed system, SM = Single melt fractions, HM = Hybrid melts. Different marker size schematically represents the volume of each melt fraction relative to the original equilibration volume.

**Figure 5:**
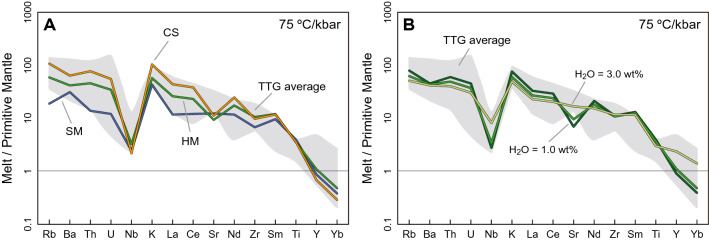
Primitive mantle normalized^33^ spider diagrams for representative accumulated melt compositions generated along the 75 °C/kbar geotherm. (**A**) Melts produced during the third MLE with a minimally water saturated protolith. (**B**) Melts produced during the third MLE considering partial melting of the protolith under fluid absent (1.0 wt% H_2_O), minimally water-saturated solidus, and water-excess conditions (3.0 wt% H_2_O). CS = Closed system, SM = Single melt fractions, HM = Hybrid melts.

High-pressure melts show high Sr/Y and La/Yb ratios that reflect equilibration in presence of garnet and absence of plagioclase, and also strong light-to-heavy REE fractionation (Fig. 4A). Conversely, low-pressure melts generated along higher geothermal gradients have lower Sr and higher Y and HREE concentrations, which reduces REE fractionation as the garnet mode diminishes (Figs. 1A and 5). In a closed system scenario, melts above ~ 30 vol% that formed along the 50 and 75 °C/kbar geotherms are equilibrated with high proportions of garnet and thus show Sr/Y, La/Yb, and (La/Yb)_N_ values higher than the average of natural TTGs (Fig. 4A,B). At low pressure, the melts produced have low Sr/Y values, which result from higher stability of plagioclase and match the signature of potassic granitoids. Taken individually, only those melt fractions generated before the first melt extraction events fall within the average TTG values of Sr/Y and La/Yb. Single melts equilibrated at relatively high temperatures along high thermal gradients shift toward lower La/Yb values, which results from a progressively more depleted source and reduction in the mode of hornblende (Fig. 4A). The observed discrepancies between natural TTGs with individual melt fractions and melts produced in a closed system appear to be resolved when mixing and accumulation of multiple melt fractions are considered (Figs. 4, 5A). Melts produced along geotherms of 50 and 75 °C/kbar have the closest match with medium- and low-pressure TTGs, producing up to ~ 40 vol% of suitable melt compositions. Similarly, the trace element composition and degree of LREE to HREE fractionation show the best correlations with the fractionation pattern of average TTG compositions for hybrid melts formed by accumulation of individual melt fractions (Fig. 5A). The changes in trace-element composition due to variations in the critical melt threshold are minimal for hybrid melts that fall in the range of average TTGs since the total melt fraction extracted from the source and the mineral proportions in the residuum are similar in all cases (Supplementary Figure 6).

The water content at the solidus also influences the stability of solid phases and thus the trace-element composition of extracted melts (Fig. 5b). With water-excess conditions, partial melting reactions rapidly reduce the mode of plagioclase, while increasing the proportion of hornblende in the residuum. Also, since the initial critical melt fraction is attained at a lower temperature, the first MLE occurs at *P–T* conditions where garnet is absent (Supplementary Figure 2). As a result, water-fluxed melting results in initial partial melts that have relatively low La/Yb ratio and high Sr concentration in comparison with melts produced under water-absent and minimally water-saturated conditions (Figs. 4, 5b). These signatures are partially modified to more fractionated patterns in hybrid melts as subsequent melt fractions are equilibrated in presence of garnet. Water-undersaturated melting, by contrast, produces highly fractionated melts with a marked negative Sr anomaly due to extended garnet stability to lower temperatures and high proportion of plagioclase in the residuum (Supplementary Figure 2).

### Implications for Archean felsic crust formation

EAT represents the average composition of Archean enriched tholeiites^16^, which is one possible source for the production of sodic TTGs^13,31^. However, different types of TTGs can be generated by partial melting of a wide variety of mafic lithologies, as previous works indicate (e.g.,^3,10,12,31,34^). For instance, a high-Fe source can stabilize more garnet at lower pressures than a high-Mg one, which leads to TTG-like melts with variable degrees of fractionation along similar geothermal gradients^12,31^. Similarly, the initial trace-element concentration of the source influences the enrichment and depletion of elements such as REE, Sr, Y, Nb, and Ta in resulting melt fractions^31^. The range of TTG compositions observed in nature is controlled by the bulk chemistry of the protolith, and not only the *P–T* conditions of melt equilibration. Hence, the results presented in this work show the potential of EAT to produce TTGs but do not rule out the possibility that other source rocks can generate them too.

Partial melting of EAT generates TTG-like melts along the three thermal gradients considered with K_2_O/Na_2_O, Mg#, and A/CNK ratios closely correlating with natural examples (Figs. 2, 3). However, we show that progressive hybridization of melt pulses, formed at sequentially higher-grade *P–T* conditions, produces the compositions that best match the geochemical characteristics observed in TTG terranes. This hybridization process is expected to occur when pulses of melt with variable composition are injected into pre-established magma chambers where melts can mix and accumulate. As such, melt hybridization not only produces compositions that match well the natural record, but also enlarges the window for TTG generation and increase the volume of continental crust generated (Fig. 2). Moreover, both water-undersaturated and water-fluxed melting can generate melts with similar trace-element patterns to average TTG compositions (Fig. 5C). This suggests that variable fluid availability at distinct crustal depths may have been important for TTG petrogenesis during the Archean^27^.

Distinctive geochemical characteristics of TTGs are best achieved when over ~ 20 vol% partial melt is produced (after the first MLE), which implies that generation of continental crust may have required either voluminous melting or accumulation of melts formed at variable metamorphic conditions (Fig. 3). Low melt fractions, by contrast, result in the generation of magmas with high K_2_O/Na_2_O ratios (Fig. 3A) that may be consistent with the less abundant occurrence of potassic granitoids^1^; yet some of them can also represent reworking of preexisting felsic crust^34,35^. Further, generation of felsic crust with ASI values within the range of natural TTGs requires melting along geothermal gradients of 50 °C/kbar or higher (Figs. 1A, 3C). Partial melts derived from less hydrated mafic crust at high- to ultra-high-pressure conditions are unlikely to generate metaluminous TTG-like melts such as those preserved in Archean cratons worldwide.

Diagnostic trace-element signatures and relatively high Mg# in natural TTGs may not necessarily require interaction of slab-derived melts with mantle peridotites (e.g.,^9^), or addition of fluids produced during slab dehydration in subduction zones (e.g.,^36,37^). As such, the mineral assemblages and mineral chemistry in the residuum during melt loss control major- and trace-element ratios in the resulting melts, and diagnostic features of TTGs can be attained at variable *P–T* conditions depending on the stable mineral assemblage (Figs. 3, 4, 5). Melt fractionation and crystallization of peritectic garnet and plagioclase during magma ascent towards the surface may account for stronger REE fractionation and typical Sr positive anomalies in natural TTGs^30,31^.

The window for the generation of all types of TTGs corresponds to melting of hydrated basaltic crust at ~ 30–60 km depth. However, voluminous melt production mainly takes place at depths of ~ 30–45 km with thermal gradients between 75 and 100 °C/kbar, most likely representing metamorphism and anatexis of overthickened crust or oceanic plateau^13,28^. These metamorphic conditions would be consistent with different non-plate tectonics regimes that may have been operational during the Archean^18^, such as mantle plumes^13,28^, crustal overturns^14,15^, and/or lithospheric peels^20,38^. Partial melting followed by melt migration toward upper crustal levels would lead to the generation of buoyant felsic crust, leaving behind high-density garnet- and pyroxene-bearing residuum (~ 3100–3500 kg m^-3^; Fig. 1B) that will become gravitationally unstable and be terminally lost into the mantle via dripping or delamination^39,40^. Moreover, minor TTG production would be expected at depths of ~ 60 km along a 50 °C/kbar geotherm; however, such crustal thickness is unlikely to be achieved in a single-lid tectonic configuration^39,18^ and is therefore consistent with evidence for the occurrence of short-lived episodes of subduction during the Archean (e.g.,^10,41,42^).

## Methods

### Phase equilibrium modeling

All petrological calculations were performed in the Na_2_O–CaO–K_2_O–FeO–MgO–Al_2_O_3_–SiO_2_–H_2_O–TiO_2_–O_2_ (NCKFMASHTO) compositional system using Theriak–Domino software^24^, and the internally consistent thermodynamic data set ds62 of Holland and Powell^43^. All calculations considered activity–composition (*a–x*) relations for tonalitic melt, augitic clinopyroxene, and clinoamphibole^44^; garnet, biotite, orthopyroxene, and chlorite^45^; muscovite–paragonite^46^; magnetite–spinel^47^; ilmenite–hematite^48^; plagioclase and K-feldspar^49^; olivine and epidote^43^. Pure phases included quartz, albite, rutile, titanite (sphene), and aqueous fluid (H_2_O). We do not consider MnO in the compositional system as the *a–x* relations used here for tonalitic melt, clinopyroxene, and clinoamphibole do not incorporate this oxide. However, even small amounts of MnO may extend the stability of garnet to lower pressures^46^, thereby affecting calculated melt compositions and the interpretation of our results. Furthermore, uncertainties on the absolute positions of assemblage field boundaries are typically less than ± 1 kbar and ± 50 °C (2σ), which is mainly a result of uncertainty on the thermodynamic properties of end-members in petrological datasets and imprecision in formulation of *a–x* relations describing mixing of end-members in solid solutions^50,51^. However, as all models employed the same thermodynamic dataset and *a–x* relations, similar errors cancel, and the results of each petrological model are thought to be relatively accurate to around 0.2 kbar and 10 °C (2σ).

The water content of the bulk composition was constrained to a ‘minimally hydrated’ scenario where 0.5 mol% H_2_O was present as a free fluid at the point of initial melting: the intersection between the solidus and the geotherm of interest (Supplementary Table 2). Consequently, phase diagrams and equilibrium calculations are only relevant for *P–T* conditions near the metamorphic paths considered. Bulk-rock Fe^3+^/ΣFe during metamorphism was fixed to that reported in the original source (~ 0.22^16^), which is within the range of estimated values for modern-day and Archean oceanic basalts ^52,53^ (Supplementary Tables 1 and 2). Phase diagrams were calculated for 50, 75, and 100 °C/kbar, as preliminary investigation of TTG magma characteristics showed that model and experimental results are most likely to match natural examples along these geotherms^3,12,13^ (Fig. 1; Supplementary Tables 3 and 6).

The results presented herein keep track of both melt composition and residuum mineralogy considering both open- and closed-system scenarios (Figs. 1B, 2; Supplementary Tables 3 and 4). For open-system conditions, melt loss was considered to occur every time a critical melt fraction of 20 vol% was reached during prograde metamorphism. This cut-off was chosen based on piston-cylinder experiments examining melt formation and segregation in amphibolites (e.g.,^21,22^). Pulses of partial melt produced at different stages during prograde metamorphism/burial along a given *P–T* path were then integrated to simulate both coalescence during ascent and injection into pre-established magma chambers. Immediately after each melt extraction event, the effective bulk composition of the system was modified by extracting three-quarters of the melt produced, while allowing the remaining melt to represent the melt connectivity threshold^22^. Each event of melt loss changes the effective bulk composition of the system and phase equilibria calculated for progressive metamorphism are therefore increasingly residual^54^.

These compositional changes were estimated using an Excel spreadsheet that takes the atomic composition of the melt phase from the Theriak-Domino output and calculates the composition of individual and accumulated melt fractions, as well as that of the solid residuum. All calculated melt volumes in Supplementary Tables 3 and 6 are relative to the original equilibration volume so that individual melt fractions decrease for consecutive MLE, whereas accumulated melt fractions increase. However, no volume constraints are required for mass balance since the composition of the produced melt fractions and the solid residuum are calculated from the ‘number’ of atoms present in each of them.

### Trace-element modeling

Trace-element modeling was performed for individual and accumulated melts that lie within 2σ from the mean major-element composition of sodic TTGs^1^. All calculations utilized weight percent modes of mineral phases in the residuum after each event of melt loss (Fig. 1B; Supplementary Table 4). The trace-element concentration in partial melting products was calculated using the modal melting Eq. ^32^:

1 $$C_{Liq} = \frac{{C_{0} }}{{D_{0} + F\left( {1 - D_{0} } \right)}}$$

2 $$C_{res} = \frac{{C_{0} D_{0} }}{{D_{0} + F\left( {1 - D_{0} } \right)}}$$

where *C*_*Liq*_ and *C*_*res*_ are the concentrations of an element in the melt and residuum, respectively; *C*_*0*_ is the concentration of that element in the source; *F* is the melt fraction in equilibrium with the solid residuum; and *D*_*0*_ is the bulk partition coefficient, which can be calculated for each element as follows:

$$D_{0} = \mathop \sum \limits_{k = 0}^{n} K_{n} X_{n}$$

Here, *K*_*n*_ is the mineral/melt partition coefficient of a given element in phase *n*, and *X*_*n*_ is the normalized modal proportion of that phase at the event of melt loss (Fig. 1B). *K*_*n*_ values for garnet, amphibole, and clinopyroxene were taken from Xiong^29^ and Bédard^28^ for the rest of phases considered (see Supplementary Table 5). The original trace-element concentration of the source corresponds to the average composition of enriched Archean mafic crust^2^ (Supplementary Table 1). Zircon was excluded from the modeling, due to its modal proportion in Archean mafic crust likely being insignificant as a result of lower average magma Zr content and polymerization state^55,56^. In addition, predicted abundances of zircon in the residuum after production of large melt volumes (i.e., > 15 vol%) would be negligible^12,57^. The initial trace-element concentration of the mafic source was modified after each melt loss event, whereas individual melt compositions formed along each geotherm were proportionally integrated to obtain the trace-element composition of corresponding hybrid melts. The resulting trace-element composition of each melt fraction is presented in Supplementary Table 6.

## Supplementary Information

Supplementary Legends.

Supplementary Tables.

Supplementary Figures.

## Data Availability

All data used for petrological modeling are provided in Supplementary Information. The software used for phase equilibrium calculations (Theriak-Domino) is available at no cost from http://www.rocks.uni-kiel.de/theriakd/html/down_en.html. Detailed results and additional source code used for PIXELMAPS calculations can be downloaded at https://github.com/jdavidhm90/Partial-melting-and-TTG-production.
