# Supplementary material for: Archean continental crust formed by magma hybridization and voluminous partial melting
Source: Sci Rep. 2021 Mar 4;11:5263. doi: 10.1038/s41598-021-84300-y (PMC7933273; doi:10.1038/s41598-021-84300-y)
Supplement: Supplementary file 1 — Supplementary Legends. [file 41598_2021_84300_MOESM1_ESM.docx]

**Supplementary Material**

**Archean continental crust formed by magma hybridization and voluminous partial melting**

**Juan David Hernández-Montenegro^1,^*****^,†^, Richard M. Palin^2^, Carlos A. Zuluaga^1^, David Hernández-Uribe^3^**

*^1^ Department of Geosciences, Universidad Nacional de Colombia, Bogotá, Colombia*

*^2^ Department of Earth Sciences, University of Oxford, South Parks Road, Oxford OX1 3AN, UK*

*^3^ Department of Earth & Environmental Sciences, University of Michigan, 1100 North University Avenue, Ann Arbor, MI 48109-1005, USA*

**Corresponding author:* [*jdavidhm90@gmail.com*](mailto:jdavidhm90@gmail.com)

**^†^** *Present address: Division of Geological and Planetary Sciences, California Institute of Technology, 1200 East California Boulevard, Pasadena, CA 91125, USA*

**Table 1.** Bulk-rock composition used for petrological modeling. Major elements correspond to the Enriched Archean tholeiite of Condie (1981) and trace elements from Martin et al. (2014).

**Table 2.** Composition of EAT under minimally water-saturated conditions at the intersection between the point of partial melting and the geotherm of interest. Values are given in wt% and as atomic proportions for Theriak-Domino input.

**Table 3.** Calculated melt compositions from phase equilibrium modeling. Oxides are reported in wt% on an anhydrous basis.

**Table 4.** Proportion of phases present in the system at specific melt volumes (closed system) and each melt loss event (open system). The density of each phase and the entire equilibration volume are included.

**Table 5.** Mineral/melt partition coefficients (K) used for trace-element modeling. Values for garnet, amphibole, and clinopyroxene are from Xiong (2006) and Bédard (2006) for the rest of phases considered.

**Table 6.** Trace-element compositions of all calculated melts.

**Figure 1.** *P–T* pseudosection for EAT showing the effects of melt fractionation on stable phase assemblages during prograde metamorphism. Each melt loss event initiates at ~20 vol.% of melt (black dashed lines) and phase relations calculated up-grade of this point are for a melt-depleted residuum. The three separated regions correspond to minimally water-saturated compositions at the intersection of the solidus and the 50, 75, and 100 °C/kbar thermal gradients (top to bottom). Calculations were performed in the Na_2_O–CaO–K_2_O–FeO–MgO–Al_2_O_3_–SiO_2_–H_2_O–TiO_2_–O_2_ (NCKFMASHTO) compositional system using Theriak-Domino software (Version 11.02.2015) (de Capitani and Petrakakis, 2010). Mineral abbreviations are based on Whitney and Evans (2010).

**Figure 2.** Calculated modal proportions (wt.%) of stable phases along a geothermal gradient of 75 °C/kbar. Each panel from top to bottom corresponds to the composition of EAT calculated at variable hydration conditions: water- undersaturation (1.0 wt.% H_2_O), minimally water-saturated (~1.6 wt.% H_2_O), and water-excess (3.0 wt.% H_2_O). Dashed lines represent melt loss events occurring at 20 vol.% melt. Boxes to the right are renormalized without melt. Calculations were performed using Theriak-Domino software (Version 11.02.2015) (de Capitani and Petrakakis, 2010). Mineral abbreviations are based on Whitney and Evans (2010).

**Figure 3.** Calculated modal proportions (wt.%) of stable phases under minimally water-saturated conditions at the intersection between the solidus and the 75 °C/kbar thermal gradient. Each panel from top to bottom corresponds to variable critical melt fractions considered for melt loss: 15 vol.%, 20 vol.%, and 25 vol.%. Dashed lines represent melt loss events. Boxes to the right are renormalized without melt. Calculations were performed using Theriak-Domino software (Version 11.02.2015) (de Capitani and Petrakakis, 2010). Mineral abbreviations are based on Whitney and Evans (2010).

**Figure 4.** Major-element compositions for melts produced along a thermal gradient of 75 °C/kbar for variable hydration conditions at the solidus: water- undersaturation (1.0 wt.% H_2_O), minimally water-saturated (~1.6 wt.% H_2_O), and water-excess (3.0 wt.% H_2_O). Colors represent the temperature range at which each melt fraction equilibrated and separated from the source. Dark and light gray regions represent the mean composition of natural Archean TTGs within ±1σ and ±2σ (Moyen, 2011), respectively.

**Figure 5.** Major-element compositions for melts produced along a thermal gradient of 75 °C/kbar under minimally water-saturated conditions at the solidus. Each panel corresponds to a different critical melt fraction: 15 vol.% (left), 20 vol.% (middle), and 25 vol.% (right). Colors represent the temperature range at which each melt fraction equilibrated and separated from the source. Dark and light gray regions represent the mean composition of natural Archean TTGs within ±1σ and ±2σ (Moyen, 2011), respectively.

**Figure 6.** Primitive mantle normalized (Sun and McDonough, 1989) spider diagram for representative accumulated melt compositions generated along the 75°C/kbar geotherm with variable critical melt fractions: 15 vol.% (blue), 20 vol.% (green), and 25 vol.% (orange).
