# Supplementary material for: Archean continental crust formed by magma hybridization and voluminous partial melting
Source: Sci Rep. 2021 Mar 4;11:5263. doi: 10.1038/s41598-021-84300-y (PMC7933273; doi:10.1038/s41598-021-84300-y)

Supplementary Figure 1

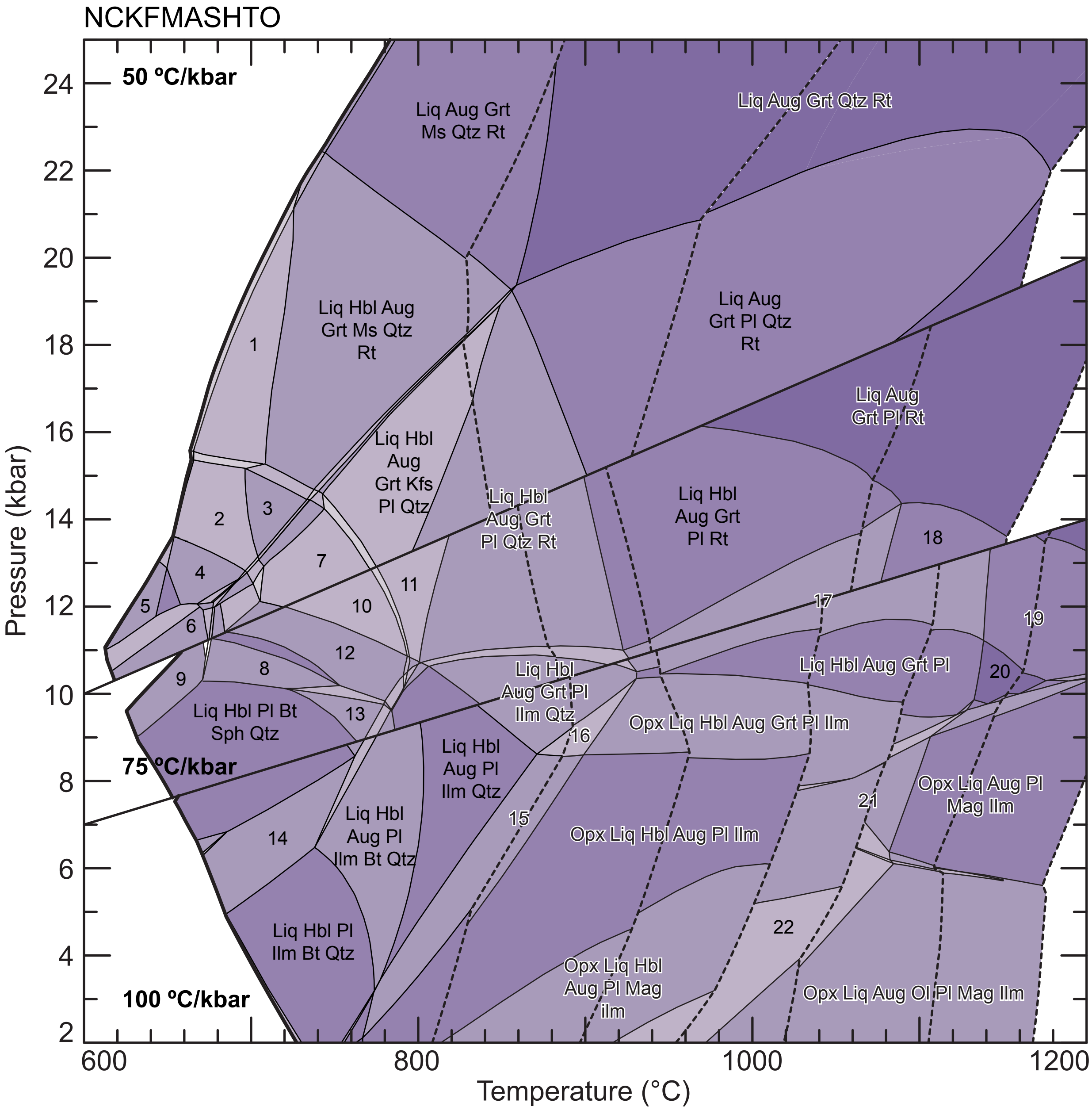

1–Liq Hbl Aug Grt Ep Ms Qtz Rt
2–Liq Hbl Aug Grt Ep Ms Sph Qtz
3–Liq Hbl Aug Grt Ms Sph Qtz
4–Liq Hbl Aug Ep Ms Sph Qtz
5–Liq Hbl Ep Bt Ms Sph Qtz
6–Liq Hbl Pl Ep Bt Sph Qtz
7–Liq Hbl Aug Grt Kfs Pl Sph Qtz
8–Liq Hbl Kfs Pl Bt Sph Qtz
9–Liq Hbl Pl Ep Bt Sph Qtz
10–Liq Hbl Aug Grt Kfs Pl Sph Qtz
11–Liq Hbl Aug Grt Kfs Pl Qtz Rt
12–Liq Hbl Aug Kfs Pl Sph Qtz
13–Liq Hbl Aug Pl Sph Qtz
14–Liq Hbl Pl Ilm Bt Sph Qtz
15–Opx Liq Hbl Aug Pl Ilm Qtz
16–Opx Liq Hbl Aug Grt Pl Ilm Qtz
17–Liq Hbl Aug Grt Pl Ilm Rt
18–Liq Aug Grt Pl Ilm Rt
19–Liq Aug Grt Pl Ilm Rt
20–Liq Aug Grt Pl Ilm
21–Opx Liq Hbl Aug Pl Mag Ilm
22–Opx Liq Hbl Aug Ol Pl Mag Ilm

Supplementary Figure 2

Water under-saturated: 1.0 wt% H_2O

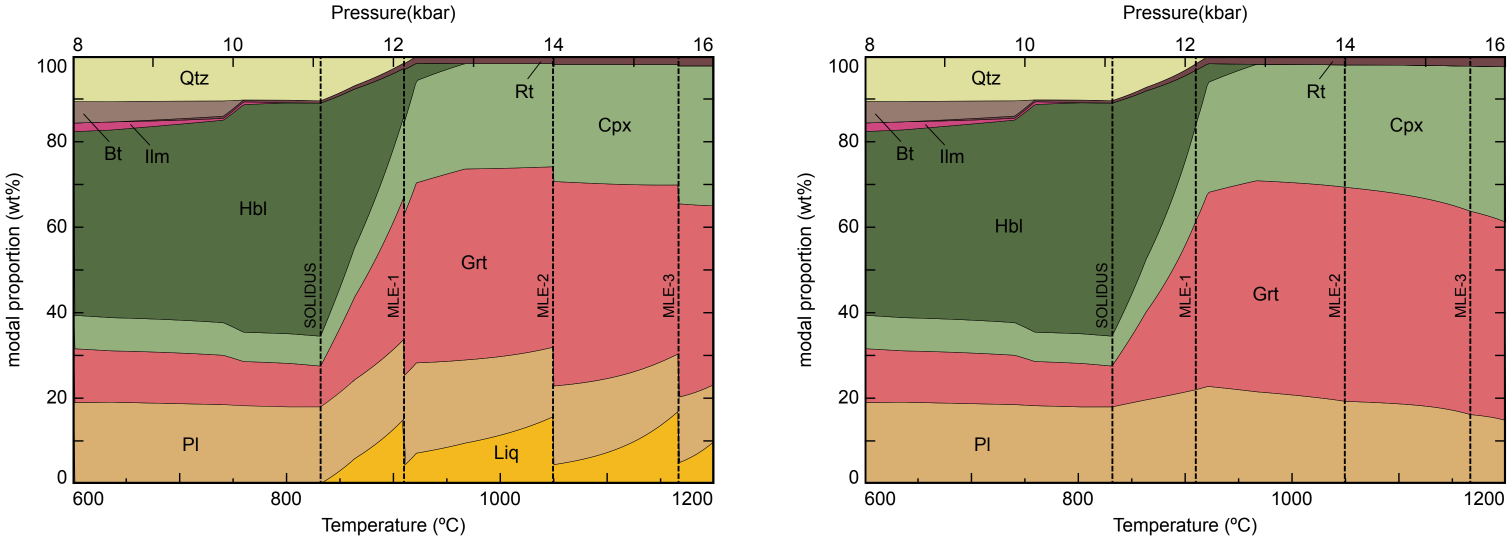

Minimally water-saturated solidus

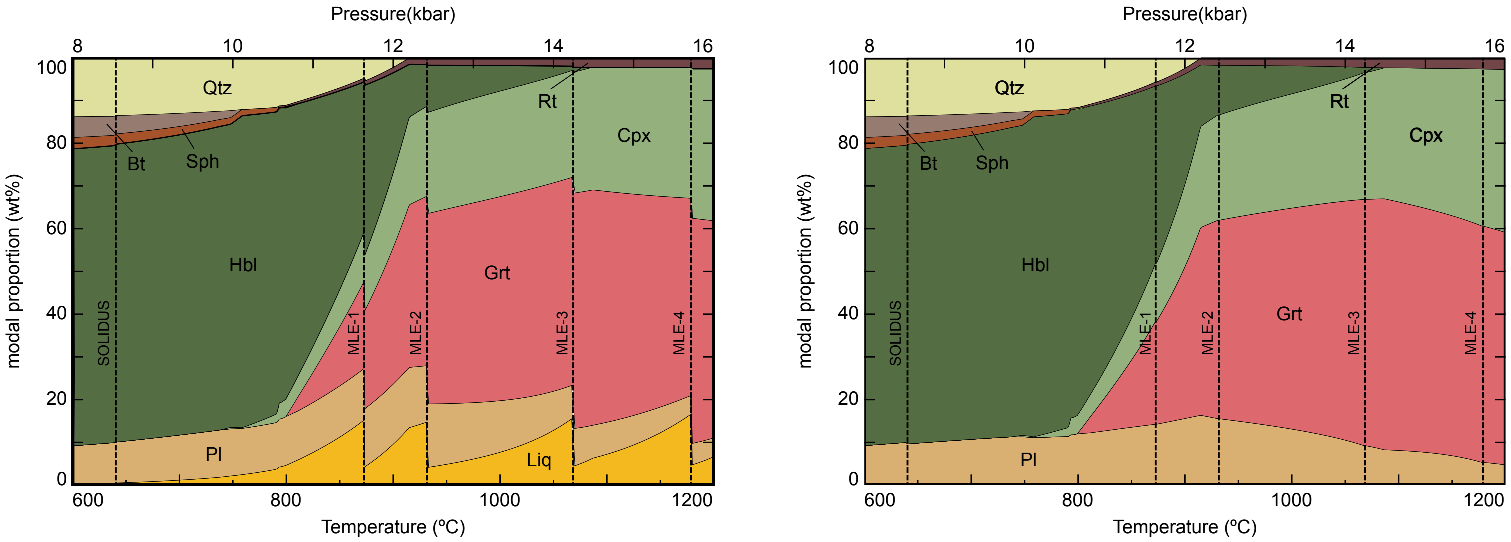

Water saturated: 3.0 wt% H_2O

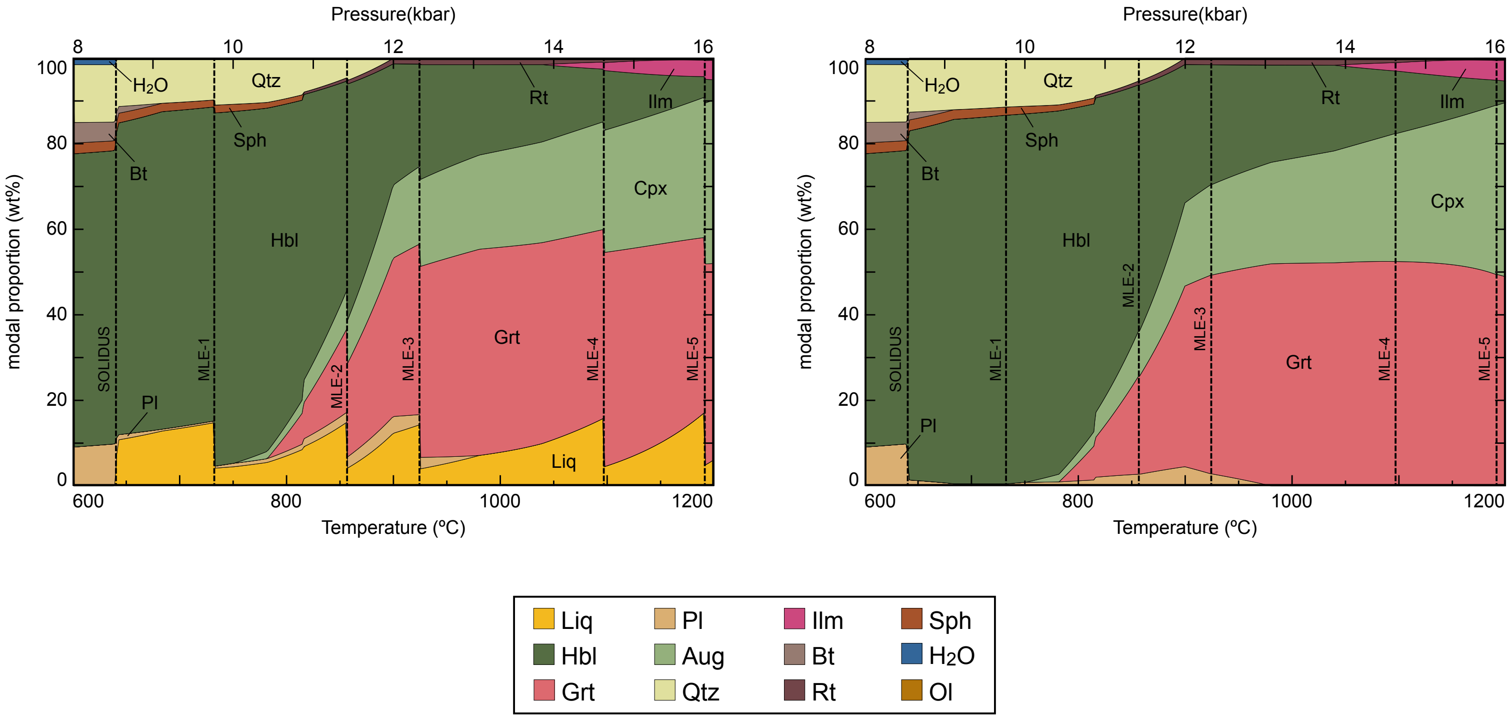

Supplementary Figure 3

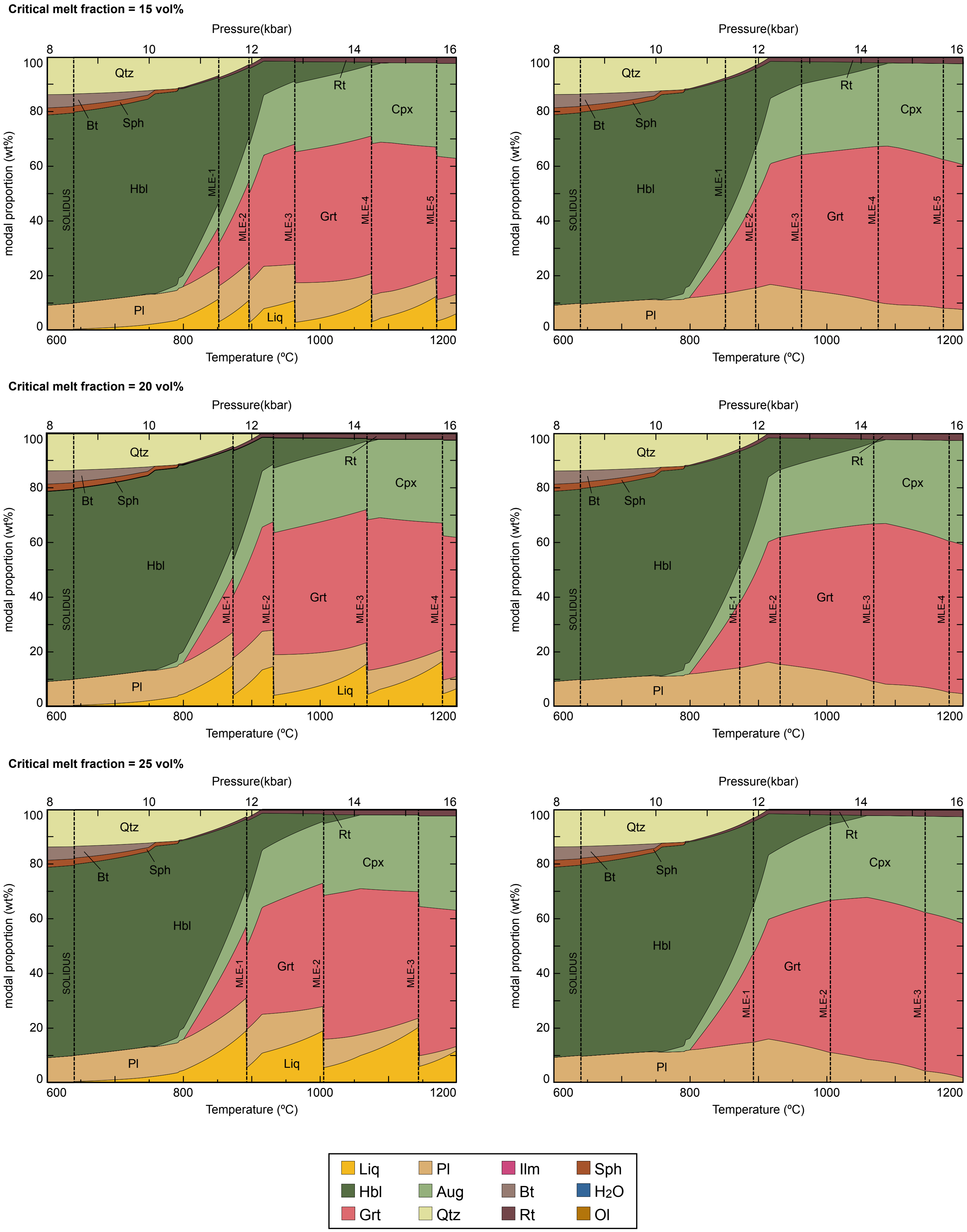

Supplementary Figure 4

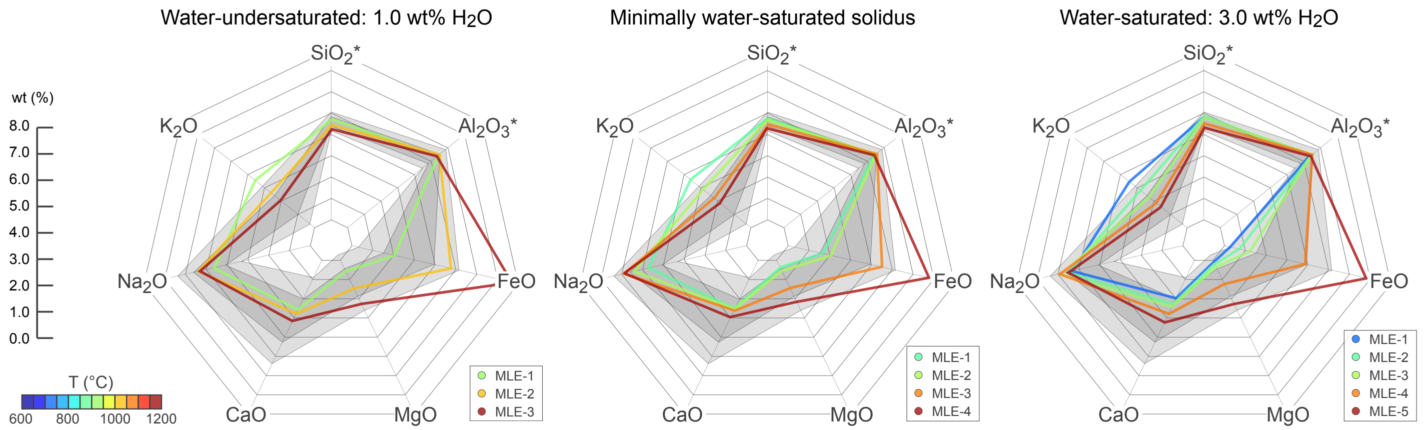

Supplementary Figure 5

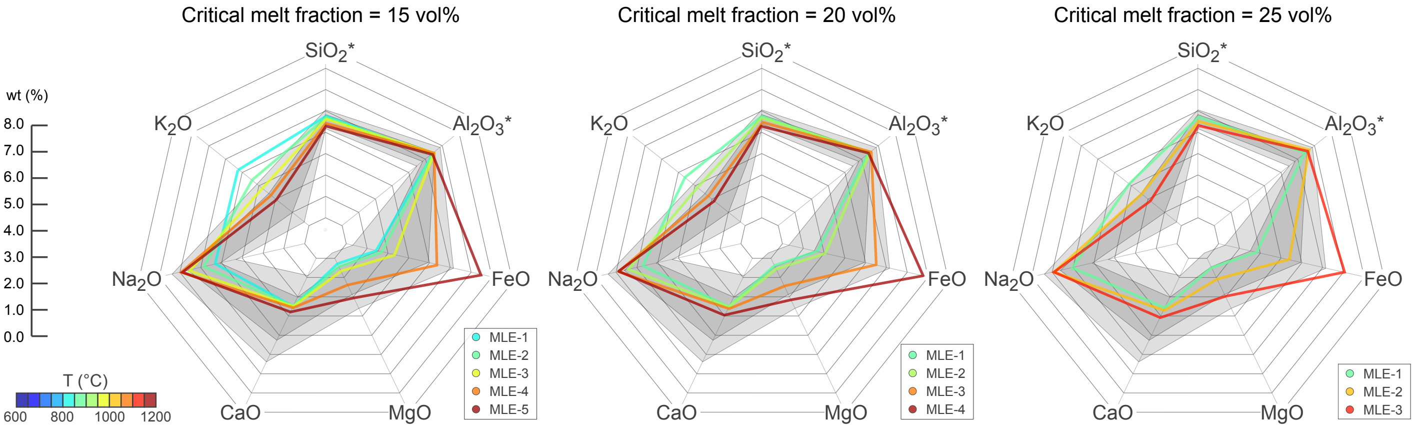

Supplementary Figure 6

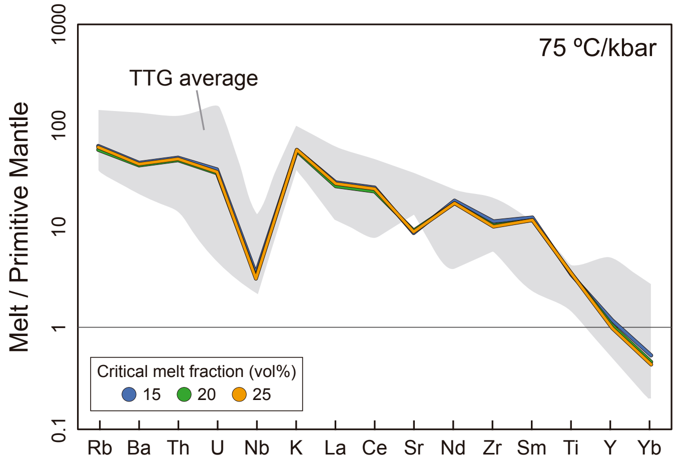

Supplement: Supplementary file 3 — Supplementary Figures. [file 41598_2021_84300_MOESM3_ESM.pdf]
